# Circulating Exosomal CircRNAs as Diagnostic Biomarkers for Chronic Coronary Syndrome

**DOI:** 10.3390/metabo13101066

**Published:** 2023-10-10

**Authors:** Xiaoyan Liu, Meili Zheng, Ruijuan Han, Ziyang Yu, Wen Yuan, Boqia Xie, Yeping Zhang, Jiuchang Zhong, Lefeng Wang, Lixia Wang, Xinming Liu

**Affiliations:** 1 Heart Center and Beijing Key Laboratory of Hypertension, Beijing Chao-Yang Hospital, Capital Medical University, Beijing 100020, China; lxycyyy@mail.ccmu.edu.cn (X.L.); zhengmeilicyh@mail.ccmu.edu.cn (M.Z.); dr.boqiaxie@mail.ccmu.edu.cn (B.X.); clay@mail.cumu.edu.cn (Y.Z.); jczhong@mail.ccmu.edu.cn (J.Z.); wanglefeng@mail.ccmu.edu.cn (L.W.); 2 Department of Cardiology, Beijing Chao-Yang Hospital, Capital Medical University, Beijing 100020, China; 3 Medical Research Center, Beijing Institute of Respiratory Medicine and Beijing Chao-Yang Hospital, Capital Medical University, Beijing 100020, China; rainbow@ccmu.edu.cn; 4 Department of Cardiology, The Second Affiliated Hospital, School of Medicine, The Chinese University of Hong Kong, Shenzhen & Longgang District People’s Hospital of Shenzhen, Shenzhen 518172, China; ruijuanhan@163.com; 5 School of Traditional Chinese Medicine, Beijing University of Chinese Medicine, Beijing 100105, China; 20210122004@bucm.edu.cn; 6 Department of Cardiology, Sinopharm Tongmei General Hospital, Datong 037003, China

**Keywords:** coronary arterial disease, chronic coronary syndrome, exosomes, circRNA, biomarker

## Abstract

Circular RNA (circRNA) has been reported to be involved in the pathogenesis of cardiovascular disease; however, it is unclear whether circRNA carried by exosomes (exos) can be used as biomarkers for chronic coronary syndrome (CCS). High-throughput sequencing was carried out in the plasma exosomal RNA of 15 CCS patients and 15 non-cardiac chest pain patients (NCCP, control group) to screen for differentially expressed circRNAs. Selected differentially expressed exo-circRNAs were further verified by real-time polymerase chain reaction in a small-sample cohort and a large-sample cohort. A total of 276 circRNAs were differentially expressed in the plasma exosomes of CCS patients, with 103 up-regulated and 173 down-regulated. Among the 103 up-regulated circRNAs, 5 circRNAs with high expression levels were selected for validation. Real time quantitative PCR of the first and second validation cohort demonstrated that exo-hsa_circ_0075269 and exo-hsa_circ_0000284 were significantly up-regulated in patients with CCS. Circulating exo-hsa_circ_0075269 and exo-hsa_circ_0000284 yielded the area under the curve values of 0.761 (*p* < 0.001, 95%CI = 0.669, 0.852) and 0.623 (*p* = 0.015, 95%CI = 0.522, 0.724) for CCS, respectively, by ROC curve analysis. In conclusion, the expression profile of circRNA in plasma exosomes of patients with CCS was significantly different from that of the control group. Plasma exo-hsa_circ_0075269 and exo-hsa_circ_0000284 have the potential to be new biomarkers for CCS.

## 1. Introduction

Coronary artery disease (CAD) remains the leading cause of morbidity and mortality worldwide despite revascularization and optimal secondary preventive treatment having been founded [1]. The concept of chronic coronary syndrome was first introduced in the “2019ESC Guidelines for the Diagnosis and Management of Chronic Coronary Syndrome” released in August 2019 [2]. CCS refers to the different developmental stages of coronary heart disease in patients with CAD other than the clinical manifestations caused by acute coronary thrombosis, and encompasses stable angina, silent ischemia, and ischemic heart disease without acute coronary syndrome. CCS is a significant contributor to cardiovascular events, such as myocardial infarction (heart attack), heart failure, and sudden cardiac death, making it a leading cause of morbidity and mortality on a global scale. Coronary angiography is the gold standard for the diagnosis of CCS. However, due to its invasiveness and high cost, it is urgent to identify suitable biomarkers for the early diagnosis of CCS, which may also contribute to the novel and beneficial therapies for CAD.

Circular RNAs (circRNAs) are a class of non-coding RNA molecules with covalently closed circular structures. Many of them are abundant, endogenous, stable, evolutionarily conserved, and exhibit cell-type-specific and tissue-specific patterns [3,4]. Recent studies have found a number of circRNAs linked to physiological and pathological development in various cardiovascular diseases, such as atherosclerosis, CAD and heart failure [5,6,7]. Due to their insusceptibility to degradation by RNA exonucleases and increased stability with significantly longer half-lives than linear RNAs [8,9], circRNAs may be potential candidates for diagnostic and prognostic biomarker for cardiovascular diseases. Notably, the stability of circRNAs may be conductive to the enrichment of circRNAs in exosomes [10]. Studies have confirmed that exosomal circRNAs can be detected in circulation and urine [11], which may contribute to finding the diagnostic relevance of exosomal circRNAs with CCS. However, the expression pattern of circulating exosomal circRNA in CCS patients, and whether it can be used as a biomarker for CCS, remains unclear. 

Hence, the aim of the study is to investigate the expression pattern of circulating exosomal circRNAs in CCS patients, and to screen for potential biomarkers for the diagnosis of CCS. It was found that, the expression profile of circRNA in plasma exosomes of patients with CCS was significantly different from that of the control group. Plasma exo-hsa_circ_0075269 and exo-hsa_circ_0000284 have the potential to be new biomarkers for CCS.

## 2. Materials and Methods

### 2.1. Study Patients

One hundred and thirty-five patients diagnosed with CCS and 83 patients with non-cardiogenic chest pain (NCCP) were included from our previous work [12]. The diagnosis of CCS is according to definition in the European Society of Cardiology (ESC) Clinical Practice Guidelines in 2019 [2], and patients with coronary artery stenosis ≥ 50% on angiography were included in this study [13]. Patients with ST-segment elevation (STEMI), non-ST-segment elevation myocardial infarction (NSTEMI), hypothyroidism, bronchial asthma, nephropathy, and malignancy have been excluded. The control group consisted of patients with NCCP who had chest pain, but normal cardiac biomarkers and no coronary stenosis as confirmed by angiography. 

**Figure 1:**
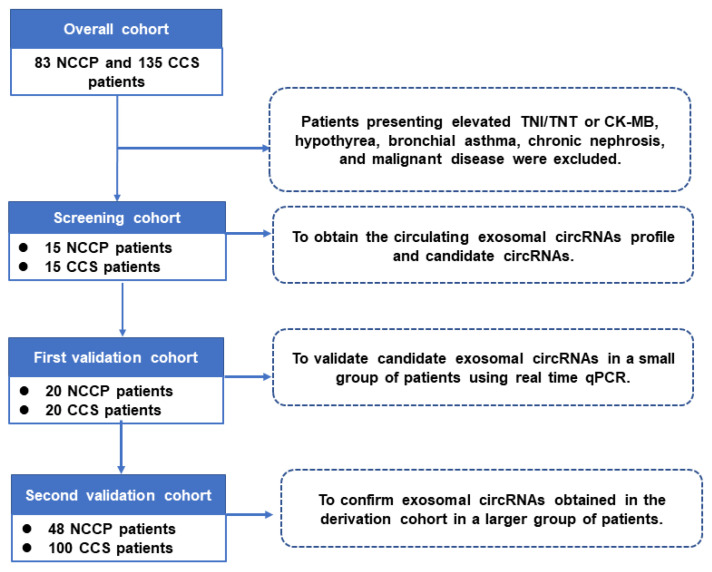
Flow chart of the present study. NCCP: non-cardiac chest pain patients; CCS: chronic coronary syndrome.

A total of 218 subjects were included in the present study and all the participants were divided into three cohorts as our previous study [12]: the sequencing cohort, including 15 CCSs and 15 NCCPs (to obtain the circulating exosomal circRNAs profile and candidate circRNAs by high-throughput sequencing); the first validation cohort, including 20 CCSs and 20 controls (to validate candidate exosomal circRNAs in a small group of patients using real-time quantitative PCR (RT-qPCR)); and the second validation cohort, including 100 CCSs and 48 NCCPs (to confirm exosomal circRNAs obtained in the first validation cohort in a larger group of patients) (Figure 1). The study was conducted in accordance with the Declaration of Helsinki, and the research protocol was approved by the Ethics Committee of Beijing Chao-Yang Hospital (2016-ke-101). Informed consent was obtained from all subjects.

### 2.2. Blood Collection, Plasma Separation, and Coronary Artery Angiography

The blood samples were collected on the following morning of admission into anticoagulant tubes containing ethylenediaminetetraacetic acid (EDTA) and centrifuged at 3000× *g* for 10 min, after which the plasma was aliquoted into cryopreservation tubes and stored at −80 °C until use. Coronary artery angiography was performed within 72 h after admission in both CCS patients and control subjects.

### 2.3. Exosomal Isolation 

The exosomes were isolated with exoEasy Maxi Kit (Qiagen Inc., 76064, Dusseldorf, Germany) following the manufacturer’s instructions. Briefly, the cryopreserved plasma was thawed in a 37 °C metal bath, centrifuged at 10,000× *g* for 15 min, and 1× volume of buffer was added. The mixture was then centrifuged at 500× *g* for 1 min. Then, 10 mL buffer was added and centrifuged at 3000× *g* for 5 min. After replacing with a new centrifuge tube, 1 mL eluate buffer was added and incubated for 1 min. Thereafter, the filtrate was collected following centrifugation at 500× *g* for 5 min. Next, the filtrate was incubated again for 1 min and centrifuged at 3000× *g* for 5 min. Finally, the filtrated exosomes were collected into a new RNase free EP tube and stored at −80 °C.

### 2.4. Exosome Identification 

The isolated exosomes were identified by transmission electron microscopy (TEM) for particle size and form, nanoparticle tracking analysis (NTA) for total amount of exosomes wand, and Western blotting for exosome specific proteins [14,15]. Western blotting of exosome marker proteins CD63 (RRID: AB_1283602) and heat shock protein 70 (HSP70) (RRID: AB_1617334) were identified as previously described [16]. 

### 2.5. Exosomal RNA Extraction

Exosome RNA were extracted using a commercial kit (Qiagen Inc., 217184, Dusseldorf, Germany) according to the manufacturer’s instructions. Briefly, 600 μL of lysis buffer were added to the extracted exosome supernatant and incubated at room temperature for 2 min. Then, the mixture was centrifuged at 3000× *g* for 5 min to separate the lysate from the debris. The supernatant was carefully collected without disturbing the pellet. Next, chloroform was added to the collected supernatant and mixed thoroughly by inverting the tube several times. The provided column was prepared by adding the mixture in batches. After centrifuging, the waste liquid was discarded. The column was washed with the provided buffer and 80% ethanol. Then, the filter membrane on the column was allowed to air-dry. Fourteen μL of elution buffer was added to the column and centrifuged to elute the RNA. Finally, the column was discarded, and the filtrate containing the purified RNA was collected and stored at −80 °C. 

### 2.6. RNA Sequencing

Exosome RNA was quantified from the OD260/280 read by a NanoDrop ND-1000 instrument. High-throughput RNA sequencing was performed at Guangzhou RiboBio Co., Ltd., Guangzhou, China. In brief, exosome RNA was purified and reverse-transcribed into cDNA, and after terminal repair, splicing, PCR, and quality inspection of the library, sequencing was performed on an Illumina Hiseq 3000 platform. Sequence data are available on the Gene Expression Omnibus (GEO; accession number: GSE106333). 

### 2.7. Real Time Quantitative PCR

The relative expression levels of exo-circRNAs were quantified using ViiA 7 Real-Time PCR System (Applied Biosystems, Foster City, CA, USA), and the forward and reverse primers included were listed in Table 1. 

### 2.8. Statistical Analysis

SPSS 24.0 software was used for statistical analysis. Continuous variables were expressed as mean ± standard deviation (x ± s). *t*-tests were used for comparison between two groups for normally distributed data. Non-normal distributed data were presented as median (25th and 75th percentile) and compared by Mann–Whitney U tests. In addition, Chi-Square test was used for the comparison of qualitative data. Differences in circRNAs levels were determined by Mann–Whitney U test. To evaluate the clinical diagnostic value of selected exo-circRNAs for CCS, receiver operating characteristic (ROC) curve and the area under the curve (AUC) were computed. AUC = 0.5 indicated no diagnostic value. ROC curve analysis was used to determine the cut-off value and corresponding sensitivity and specificity. All statistical tests were two-tailed, and *p*-values < 0.05 represented statistical significance.

## 3. Results

### 3.1. Identification of Exosomes

**Figure 2:**
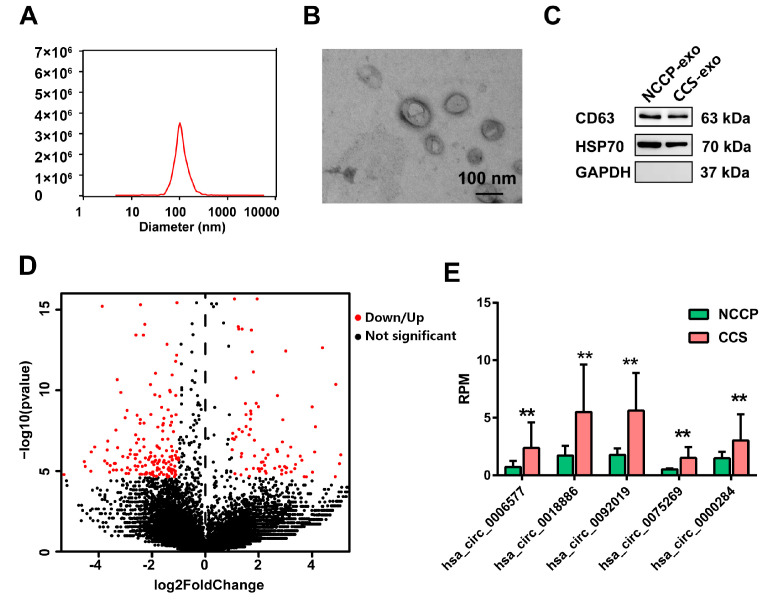
Exosome identification for sequencing plasma samples by Western blotting and circulating exosomal circRNAs sequencing in CCS and NCCP controls (**A**) nanoparticle tracking analysis (**B**) and TEM (**C**), respectively. (**D**) the volcano plot of substantially differentially expressed (|log2fold change| > 1 and q-value < 0.001) circRNAs in CCS patients and NCCP controls; (**E**) 7 up-regulated circRNAs in CCS were selected for the first validation cohort. CCS, chronic coronary syndrome; NCCP, non-cardiogenic chest pain; TEM: transmission electron microscopy; HSP70, heat shock protein 70; RPM, reads per million mapped reads. ** *p* < 0.01.

The size of the extracted particles ranged from 30 to 200 nm, and the peak value was at 100 nm, which is consistent with the particle size characteristics of exosomes (Figure 2A). Morphologies under TEM showed that the vesicles were round in shape and had a bilayer membrane structure (Figure 2B). Western blot results displayed that the vesicles expressed CD63 and HSP70, which are exosome specific markers, but did not express GAPDH, further confirming that the extracted vesicles were exosomes (Figure 2C).

### 3.2. Sequencing Profiles of Circulating Exosomal circRNAs

Fifteen CCS patients and 15 NCCP subjects were included for RNA sequencing. For the baseline characteristics of the sequencing samples, please see our previously published data [12]. The assessment of clinical characteristics revealed no statistical difference between CCS patients and the controls in terms of age, sex, hypertension, diabetics, family history, heart rate, systolic blood pressure, diastolic blood pressure, body mass index, or laboratory data. 

Using |log_2_fold change| > 1, q-value < 0.001 as the cut-off, 276 differentially expressed circRNAs (103 up-regulated and 173 down-regulated) were detected in CCS plasma exosomes compared to that of the NCCP group (Figure 2D). Among the differentially expressed circRNAs, 59 circRNAs were annotated in the circBase. 

To identify biomarkers suitable for clinical application, five circRNAs (hsa_circ_0006577, hsa_circ_0018886, hsa_circ_0092019, hsa_circ_0075269, and hsa_circ_0000284) were chosen as candidate circRNAs for further validation based on the following criteria: annotated in the circBase database (http://circrna.org/, accessed on 10 October 2020), significant differential expression, and high expression in the samples (Figure 2E). 

### 3.3. Validation of Candidate Exosomal circRNAs in the First Validation Cohort

To identify potential exosomal circRNAs that can be served as CCS diagnostic biomarkers, the five candidate exosomal circRNAs were detected in the first validation cohort, which includes 20 CCSs and 20 NCCPs, using RT-qPCR. For the clinical characters of the first validation cohort, please see our previously published data [12]. The analysis of clinical features showed that patients with CCS were older and had significantly increased level of leukocyte and neutrophil than the NCCP controls. There were no differences between the two groups in other physical data (including age, sex, smoker, drinker, cardiac function, heart rate [HR], systolic blood pressure [SBP], diastolic blood pressure [DBP], and body mass index [BMI]), historical data (including history of hypertension, diabetes, and family history), and laboratory data (except for leukocyte and neutrophil counts).

**Figure 3:**
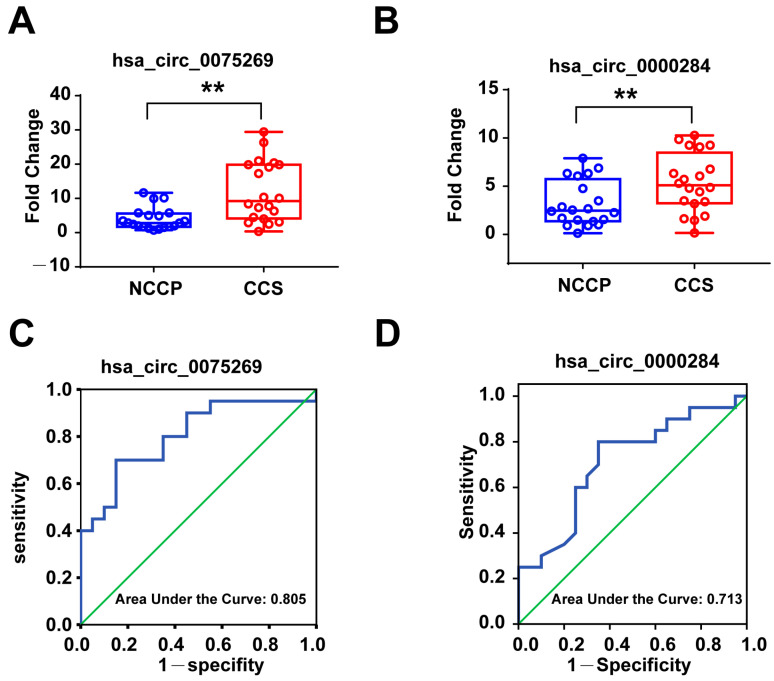
Circulating exosomal hsa_circ_0075269 and hsa_circ_0000284 in the first validation cohort. (**A**) RT-qPCR analysis of expression of exosomal hsa_circ_0075269; (**B**) RT-qPCR analysis of expression of exosome hsa_circ_0000284; (**C**) receiver operator characteristic (ROC) analysis of exosome hsa_circ_0075269, area under the curve (AUC) = 0.805 (*p* = 0.001, 95%CI = 0.667, 0.943); (**D**) ROC analysis of exosomal hsa_circ_0000284, AUC = 0.713 (*p* = 0.021, 95%CI = 0.550, 0.875. CCS, chronic coronary syndrome; NCCP, non-cardiogenic chest pain. ** *p* < 0.01.

The RT-qPCR results showed that the expression of hsa_circ_0018886 was too low to be detected in most of the samples. In addition, there was no difference in hsa_circ_0006577 and hsa_circ_0092019 between the two groups, respectively. Notably, the expression level of hsa_circ_0075269 and hsa_circ_0000284 were significantly increased in CCS patients compared with NCCP group (Figure 3A,B), which was consistent with the RNA sequencing results.

### 3.4. Diagnostic Power of Plasma Exosomal circRNAs in the First Validation Cohort

The diagnostic accuracy and discriminatory power of hsa_circ_0075269 and hsa_circ_0000284 for patients with CCS from NCCP was determined using receiver operator characteristic (ROC) analysis and illustrated by area under the curve (AUC) (Table 2). The ROC curve analysis of the circulating exosomal hsa_circ_0075269 showed an AUC = 0.805 (*p* = 0.001, 95%CI = 0.667, 0.943) for CCS patients. Using 6.05 as the cut-off value, the sensitivity and specificity were 70% and 85%, respectively, and the positive predictive value (PPV) and negative predictive value (NPV) were 65% and 85%, respectively (Table 2). Moreover, circulating exosomal hsa_circ_0000284 showed diagnostic value for CCS patients with AUC value of 0.713 (*p* = 0.021, 95%CI = 0.550, 0.875) (Figure 3C,D). Using 3.02 as the cut-off value, the sensitivity and specificity were 80% and 65%, respectively, and the PPV and NPV were 65% and 85%, respectively (Table 2).

### 3.5. Validation of Derived Circulating Exosomal circRNAs in the Second Validation Cohort

To confirm exosomal circRNAs obtained in the first validation cohort in a larger group of patients, RT-qPCR was performed on the second validation cohort, which includes 100 CCS patients and 48 NCCP controls. For their clinical characteristics, please see Table 2 of our previously published data [12]. The proportion of age, gender, smoking, and left ventricular end diastolic diameter (LVEDD) were larger in the CCS group than the NCCP group. The assessment of clinical characteristics indicated no significant difference between the two groups for most of the clinical variables measured, except that CCS patients had significantly higher plasma levels of leukocyte and lymphocyte counts (*p* = 0.011, 0.039, respectively), alanine transaminase (ALT) (*p* = 0.003), low-density lipoprotein (LDL) (*p* = 0.002), high-density lipoprotein (HDL) (*p* = 0.004), glycosylated hemoglobin (HbA1C) (*p* = 0.006), and K+ (*p* = 0.012). 

**Figure 4:**
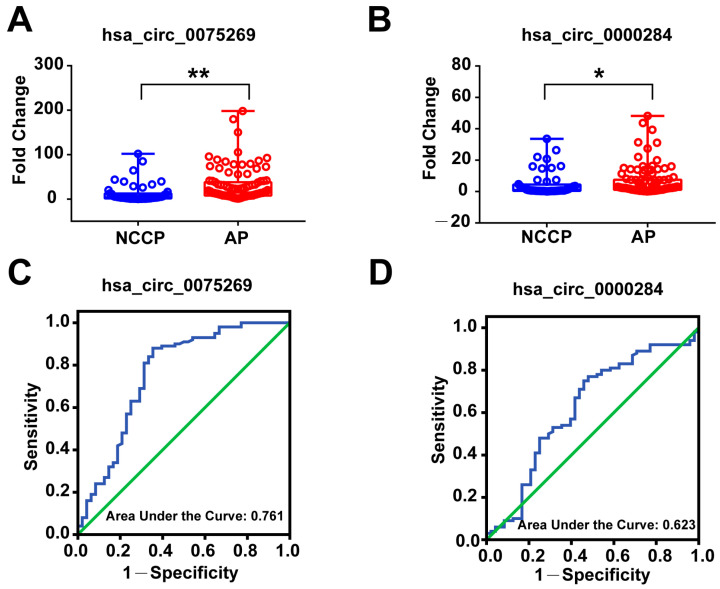
Circulating exosomal hsa_circ_0075269 and hsa_circ_0000284 in the second validation cohort. (**A**) RT-qPCR analysis of expression of exosome hsa_circ_0075269; (**B**) RT-qPCR analysis of expression of exosomal hsa_circ_0000284; (**C**) receiver operator characteristic (ROC) analysis of exosome hsa_circ_0075269, area under the curve (AUC) = 0.761 (*p* < 0.001, 95% = 0.669, 0.852); (**D**) ROC analysis of exosomal hsa_circ_0000284, AUC = 0.623 (*p* = 0.015, 95%CI = 0.522, 0.724). CCS, chronic coronary syndrome; NCCP, non-cardiogenic chest pain. ** *p* < 0.01; * *p* < 0.01.

As expected, the expression of hsa_circ_0075269 (14.78 (7.96, 38.06)) (Figure 4A) and hsa_circ_0000284 (2.52 (1.14, 7.53)) (Figure 4B) in plasma exosomes of CCS patients were significantly higher than that of NCCP patients, which was consistent with the results from sequencing and first validation cohorts.

### 3.6. Diagnostic Power of Plasma Exosomal circRNAs in the Second Validation Cohort

To investigate the diagnostic value of circulating exosomal hsa_circ_0075269 and hsa_circ_0000284 in CCS, AUC values for their respective ROC curves were obtained from the second validation cohort (Table 3). The ROC curve analysis of circulating exosomal hsa_circ_0075269 showed an AUC = 0.761 (*p* < 0.001, 95% = 0.669, 0.852) (Figure 4C) for CCS patients. Using 5.66 as the cut-off value, the sensitivity and specificity were 70% and 85%, respectively, and the PPV and NPV were 85% and 65%, respectively. On the other hand, hsa_circ_0000284 showed a weaker discriminatory power for CCS patients with AUC = 0.623 (*p* = 0.015, 95%CI = 0.522, 0.724) (Figure 4D). Using 1.97 as the cut-off value, the sensitivity and specificity were 75% and 54.2%, respectively, and the PPV and NPV were 63% and 56%, respectively.

## 4. Discussion

The present study was designed to investigate the expression pattern of circulating exo-circRNAs in CCS patients, and to screen for potential biomarkers for the diagnosis of CCS. Our current findings demonstrated that the expression pattern of circRNAs in the plasma exosomes of patients with CCS was significantly different from that of the control group, with 103 up-regulated and 173 down-regulated; plasma exo-hsa_circ_0075269 and exo-hsa_circ_0000284 were significantly up-regulated in patients with CCS and have the potential to be new diagnostic biomarkers for CCS.

CAD and its major complication are the leading causes of cardiovascular morbidity and mortality, and it has cost a high economic burden of the national healthcare system [17]. Accumulating evidence suggests that the application of some biomarkers reflecting specific pathophysiological features, such as oxidative stress, emergency vascular inflammation, platelet activation, plaque destabilization and rupture, and markers of necrosis, may improve the identification and risk stratification of coronary lesions. However, there are still no validated biomarkers to discern the occurrence of CCS in the CAD population. 

Recently, the roles of exosomes and circRNAs in CAD have received increasing attention. Exosomes are the smallest extracellular vesicles that are secreted into the extracellular environment upon fusion of intracellular multivesicular bodies with the plasma membrane. Recent research is highlighting the role of exosomes in intercellular communication in cardiovascular microenvironment [18,19,20]. Exosomes released from multiple cells, including potential valuable biological information, for the development and progression of CAD, like stem cells, endothelial cells, smooth muscle cells, cardiomyocytes, adipose cells, and platelets [18]. A previous study revealed that the serum exosomes of acute coronary disease (ACS) patients showed a high level of miR-208a compared to the healthy controls. Within a 1-year follow up, patients with low miR-208a expression were more significantly correlated with a lower mortality rate than those with high miR-208a expression (3.3% vs. 11.0%, *p* < 0.05) [21]. More importantly, the study found that the sensitivity of serum miR-208a was inferior to that of exosome miR-208a. Similar to microRNAs, circRNAs in circulating exosomes have a more stable structure than that in plasma.

In the past 5 years, high-throughput sequencing has facilitated the identification of circRNAs with a potential role in cardiovascular diseases (CVD) [22]. The closed loop-ended structure of circRNAs were more stable than linear transcripts because of the special structure protecting them from degradation by exoribonucleases. Their applicability as clinical biomarkers of CVD also have been explored [23,24,25]. Zhao et al. first investigated the whole peripheral blood in circRNA profile of 12 CAD patients and 12 controls to determine its correlation with the severity of CAD, and to test the potential of circRNA as a diagnostic biomarker of CAD [26]. circRNAs are important information in exosomes and can be protected by exosomes, making them considerably less susceptible to degradation. Studies have shown that exosomal circRNAs can be used as diagnostic and prognostic biomarkers for some tumor diseases [27,28]. Therefore, we speculated that exosome cirRNAs may also be the potential biomarkers of CCS. 

Using high-throughput sequencing, 103 up-regulated and 173 down-regulated plasma exo-circRNAs were found in CCS group, and 59 circRNAs were annotated in the circBase database. Generally, high-throughput sequencing has fewer samples, and it may involve a false positive rate, thus five significantly differentially expressed exosome circRNAs were selected for validation by RT-qPCR in the first validation cohort and validation cohort. The results showed that circulating exosomal hsa_circ_0075269 and hsa_circ_0000284 were significantly up-regulated in the CCS group, both in the first and the second validation cohort. 

Hsa_circ_0075269 is located on chromosome 5 with the full length of 288nt. The field of exosome circRNAs is quite a new area, and we have not yet found any definite evidence to demonstrate the function in tissues of hsa_circ_0075269. The mechanism in CAD needed to be further explored. Hsa_circ_0000284 is located on chromosome 11 with the full length of 1099nt, which is associated with tumor growth, migration, invasion, and angiogenesis [29,30,31]. A validation study showed that serum hsa_circ_0000284 is also associated with carotid plaque rupture and stroke [32]. Sun et al. have reported that the expression levels of hsa_circ_0000284 in peripheral blood leukocytes in CAD cases were higher than those in non-CAD subjects (all *p* < 0.05), which is consistent with our research [33].

Exo-hsa_circ_0075269 and exo-hsa_circ_0000284, may have potential biological functions and roles in CCS. However, it is important to note that the specific functions of these circRNAs in CCS have not been extensively studied, and their roles in cardiovascular disease or other relevant conditions are still largely unknown. Previous studies have shown that circRNAs can function as regulators of gene expression by acting as microRNA sponges, interacting with RNA-binding proteins, or modulating alternative splicing. These mechanisms suggest that circRNAs may have regulatory roles in various biological processes, including cell proliferation, apoptosis, inflammation, and angiogenesis, which are relevant to CCS. It is worth mentioning that the field of circRNA research is relatively new, and studies investigating their roles in specific cardiovascular diseases are still limited. Therefore, further research is needed to elucidate the functions and roles of exo-hsa_circ_0075269 and exo-hsa_circ_0000284 in CCS.

The present study also has limitations. First, the small sample size of 15 patients in each group of the screening cohort may provide initial insights, but it has limitations in statistical power. With a small sample size, the study may struggle to detect small or moderate effects between the CCS and NCCP groups. Larger sample sizes should be considered in future studies to validate and strengthen the findings. Second, as a single-center study, the subjects were geographically concentrated, and it remains doubtful whether other regions can also detect the same circRNAs profile. Third, as the expression of circRNAs was not detected at multiple time points, a dynamic relation between circRNAs and CCS cannot be determined. The conclusions of this study require further verification in larger and more diverse cohorts. Lastly, while exosome circRNAs hold promise as biomarkers, several limitations need to be addressed, including stability, reproducibility, and sensitivity. The standardization of protocols, rigorous validation, and comparison to existing biomarkers are necessary steps to establish the clinical utility of exosomal circRNAs as biomarkers in various diseases, including CCS.

## 5. Conclusions

In summary, we explored and analyzed, for the first time, the expression pattern of circulating exosome circRNAs in patients with CCS. Circulating exosome hsa_circ_0075269 and hsa_circ_0000284 are up-regulated in patients with CCS and have the potential to be new biomarkers for CCS diagnosis. The diagnostic value of hsa_circ_0075269 are relatively higher than hsa_circ_0000284, making hsa_circ_0075269 a probable tool in the diagnosis of CCS. Given the relatively small sample size, further validation in larger and more diverse cohorts are needed, and more investigations are required to define the physiological functions and underlying mechanisms by which these cricRNAs modulate the structural and functional remodeling of the heart in the progression of CCS.

## Figures and Tables

**Table 1:** Primer sequence information of RT-qPCR.

Gene	Primer Sequence
hsa_circ_0006577-Forward Primer	5′-CGCTGCCTAAGGAAATCGG-3′
hsa_circ_0006577-Reverse Primer	5′-TATGGCTGAGGACCAGTTGTG-3′
hsa_circ_0018886-Forward Primer	5′-AAGATTGAGCAAGCACAGCG-3′
hsa_circ_0018886-Reverse Primer	5′-CACATCCAGACCCTGAGATACC-3′
hsa_circ_0092019-Forward Primer	5′-GATCTTTACGGCAGGCATCG-3′
hsa_circ_0092019-Reverse Primer	5′-CACAGTGAACTCTGCCCGCT-3′
hsa_circ_0075269-Forward Primer	5′-TGATGGTGGCAAGGAGACAG-3′
hsa_circ_0075269-Reverse Primer	5′-CTCAGGCTCATAGAACTCGCTTA-3′
hsa_circ_0000284-Forward Primer	5′-TCCTGTTCGGCAGCCTTAC-3′
hsa_circ_0000284-Reverse Primer	5′-GACTTGTGAGGCCATACCTGTAG-3′
hsa-18S-Forward Primer	5′-CGCTCGCTCCTCTCCTACTT-3′
hsa-18S-Reverse Primer	5′-CGGGTTGGTTTTGATCTGATAA-3′

**Table 2:** Sensitivity and specificity of circulating exosomal circRNAs in patients with NCCP and CCS patients in the first validation cohort.

Exo-circRNAs	AUC	95%CI	*p* Value	Sensitivity	Specificity	Youden Index	Cut Off Value	PPV	NPV
has_circ_0075269	0.805	0.667–0.943	0.001	0.70	0.85	0.55	6.05	0.65	0.85
hsa_circ_0000284	0.713	0.550–0.875	0.021	0.80	0.65	0.45	3.02	0.80	0.65

Exo-circRNAs, exosomal circular RNAs; AUC, area under the ROC curve; NCCP, non-cardiogenic chest pain; CCS, chronic coronary syndrome; PPV: positive predictive value; NPV: negative predictive value.

**Table 3:** Sensitivity and specificity of circulating exosome circRNAs in patients with NCCP and CCS patients in the validation cohort.

Exo-circRNAs	AUC	95%CI	*p* Value	Sensitivity	Specificity	Youden Index	Cut Off Value	PPV	NPV
has_circ_0075269	0.761	0.669–0.852	<0.001	0.88	0.646	0.526	5.66	0.85	0.65
hsa_circ_0000284	0.623	0.522–0.724	0.015	0.75	0.542	0.292	1.97	0.63	0.56

Exo-circRNAs, exosomal circular RNAs; AUC, area under the ROC curve; NCCP, non-cardiogenic chest pain; CCS, chronic coronary syndrome; PPV: positive predictive value; NPV: negative predictive value.

## Data Availability

Sequence data are available on the Gene Expression Omnibus (GEO; accession number: GSE237983). Other data presented in this study are available upon request from the corresponding author. The data are not publicly available due to privacy.
